# Therapeutic Patient Education and Eating Habits: A Challenge in Caries Disease

**DOI:** 10.3390/dj12100322

**Published:** 2024-10-08

**Authors:** Marjorie Zanini, Mélodie Clerc, Sylvie Azogui-Levy, Annabelle Tenenbaum

**Affiliations:** 1 Dental School, University of Paris Cité, F-75006 Paris, France; marjorie.zanini@u-paris.fr (M.Z.); sylvie.azogui-levy@u-paris.fr (S.A.-L.); 2 Department of Restorative Dentistry, Pitié Salpetrière Hospital APHP, F-75013 Paris, France; 3 URP 2496 Orofacial Pathologies, Imaging and Biotherapies, University of Paris Cité, F-92120 Montrouge, France; 4 Department of Periodontics, Rothschild Hospital APHP, F-75012 Paris, France; 5 Laboratory of Molecular Oral Physiopathology, UMR 1138, University of Paris Cité, F-75006 Paris, France; 6 Department of Dental Public Health, Pitié Salpetrière Hospital APHP, F-75013 Paris, France; 7 Laboratoire Educations et Promotion de la Santé (LEPS—UR 3412), Université Sorbonne Paris Nord, F-93430 Villetaneuse, France

**Keywords:** dental caries, risk factor, dietary habits, therapeutic patient education

## Abstract

**Dietary Factors and Oral Health:** Risky dietary behaviors, particularly excessive sugar consumption, significantly contribute to dental caries. Dental practitioners are tasked with detecting and managing these behaviors to effectively treat caries and prevent recurrences. Although dietary assessment tools exist to identify such behaviors, they have limits. Furthermore, traditional methods, focused on information dissemination and advice, often fall short in promoting sustainable changes in patient behavior toward oral health. From our perspective, there is a necessity to integrate educational approaches with therapeutic management for enhancing the ability current and future dental practitioners to effectively care for their patients’ oral health needs. **Discussion**: Specific educational models have been developed for patients with chronic diseases, through Therapeutic Patient Education (TPE), defined as helping patients acquire or maintain the skills they need to best manage their lives with a chronic disease. **Future Directions**: By incorporating TPE into dental practice, oral health professionals can empower patients to take control of their eating habits and reduce their risk of caries disease. This holistic approach addresses both the carious lesions and underlying causes of tooth decay, leading to better oral health outcomes for patients.

## 1. Introduction

As mentioned in the Global Burden of Disease Study, despite collective prevention efforts, dental caries remains a pervasive issue affecting individuals of all ages, with untreated carious lesions being particularly prevalent among permanent teeth [1,2]. Dental caries arises from the demineralization of enamel and dentine, the hard tissues of the teeth, caused by organic acids. These acids are produced by dental plaque’s bacteria through the anaerobic metabolism of dietary sugars. This leads to a reduction in dental plaque pH. When the pH reaches the critical pH of 5.5, it enhances the solubility of calcium hydroxyapatite in dental hard tissues and results in demineralization. Consequently, sugar consumption emerges as a pivotal factor in the initiation and progression of caries. Moreover, numerous studies have established a positive association between the number of Decayed, Missing, Filled Teeth (DMFT) and the frequency of sugar intake [3,4,5,6]. However, the relationship between caries and sugar consumption is intricate, influenced by factors such as the timing and nature of sugar ingestion, as well as food texture [7]. For instance, sweetened beverages exhibit high cariogenicity, while the interplay between consumed foods can impact dental plaque pH levels [3,8,9,10,11,12,13]. Protecting factors, notably fluoride exposure, also exert influence, with fluoridated toothpaste helping to mitigate the association between sugar intake and DMFT [3,14].

Contemporary caries management entails the assessment and rebalancing of risk factors to stabilize the disease and prevent new caries lesions. Unlike other caries risk factors, dietary factors necessitate comprehensive evaluation, with a particular emphasis on assessing dietary behaviors. However, in the face of risky dietary habits, therapeutic approaches often rely on informational interventions and advice, which may fall short of promoting sustainable changes.

The aim of this article is to propose a shift towards an educational approach in the management of dental caries among adult patients, particularly when risky dietary behaviors have been identified.

## 2. Dietary Factors and Oral Health

Behavior is a critical factor influencing health status. As Gaston Godin posits, “health-related behavior is an action performed by an individual that has a positive or negative influence on his or her health” [15]. Thus, a specific behavior is primarily an observable action linked to health from a health perspective and possesses a social dimension associated with the individual’s life context. In the realm of oral health, certain behavioral factors, such as oral hygiene and dietary habits, represent observable actions that can yield either positive (protective factor) or negative (risk factor) consequences for oral health. It is the dental practitioner’s responsibility to identify and reinforce positive behaviors or assist patients in mitigating negative ones.

Numerous tools have been proposed to identify and characterize risky dietary behavior in the context of caries disease. The nine most commonly used tools are outlined in Table 1.

These tools come in two forms: questionnaires that can be completed by either the practitioner (hetero-questionnaire) or the patient (self-questionnaire), and food diaries completed by the patients themselves. The decision to utilize these tools ultimately rests with the dental practitioners, as there are currently no official guidelines mandating their use. While these questionnaires all incorporate dietary assessments aimed at identifying cariogenic behavior, they may also exhibit several limitations and biases that practitioners must be mindful of.

Certain questionnaires provide only a brief, subjective, and imprecise assessment. What exactly do the terms “high frequency/amount” in the International Caries Classification and Management System^™^ (ICCMS^™^), in the CariesCare International questionnaire, and “frequent snacks” in the Caries Management By Risk Assessment^®^ (CAMBRA^®^) questionnaire refer to [16,18,21]? The frequency of snacking and the amount are left to the discretion of the dental practitioner, with no threshold value. Furthermore, these questionnaires do not consider the composition of meals and the interactions between the food items. Thus, while these questionnaires may be useful in identifying risky dietary behaviors, they may not be sufficient for characterizing them. The Diet Assessment of Caries Tool and the Nutrition Questionnaire for Dental Caries Risk Factors, as well as the Non-Nutritive Sweetener Food Frequency Questionnaire (NNS Questionnaire), provide more precise assessment of the diet. For example, the Diet Assessment of Caries Tool focuses on the nature and frequency of foods consumed, while the Nutrition Questionnaire for Dental Caries Risk Factors analyzes the consumption of sugary foods and drinks throughout the day, along with milk consumption [20,22,23]. Similarly, the NNS Questionnaire specifically evaluates the intake of sugary ultra-processed foods and foods containing synthetic sugars, although it does not assess meal composition. Questionnaires containing detailed lists of sweet products, such as the Nutrition Questionnaire for Dental Caries Risk Factors and the NNS Questionnaire, are more relevant as they allow for the precise identification of cariogenic products consumed and their interactions.

However, these questionnaires are often perceived as time-consuming for both the patient and dental practitioner and are therefore generally reserved for patients suspected of having high-risk eating habits. Another tool that could be useful in such situations is a food diary. However, it is best used over several days to provide a more comprehensive assessment of an individual’s eating behavior [14].

Adapting assessment questionnaires to focus on the diets specific to each population is crucial for obtaining the most reliable results [25]. Although each of the questionnaires has positive aspects, none offers an educational approach.

## 3. Discussion

The first step in a comprehensive patient management strategy is to identify cariogenic risk factors, for which the dental practitioner can use the tools described above. Subsequently, it necessitates adopting an educational posture, offering support to the patient in modifying his/her behavior. Risky dietary behaviors could be perceived as ”manageable” through active patient involvement. This is of particularly importance as caries disease is chronic and requires long-term follow-up. Conventional educational approaches based solely on the dissemination of information and advice have shown to lead to limited efficacy in promoting sustainable behavioral changes in oral-health patients. Although they may increase patient knowledge, they rarely result in sustainable behavioral changes. Various tools have been tested to improve communication with the patient, with the aim of improving health behaviors and attitudes. For example, Miller and Rollnick proposed “motivational interviewing”, the aim of which is to “better understand the need for behavior change from the point of view of the person concerned. This can be achieved through techniques such as empathy, highlighting discrepancies, managing resistance to change and efforts to improve self-efficacy” [26]. Studies have proved its efficacy in improving oral health outcomes [27,28,29,30]. Additionally, it has already been successfully integrated into daily practice for initial periodontal care [30] and in cariology within pediatric dentistry [31,32,33].

In addition to patient communication and support for health behaviors, educational models have been developed specifically for patients with chronic diseases notably therapeutic patient education (TPE). According to the WHO, in the field of Prevention of Chronic Diseases, Therapeutic Patient Education is defined as “helping patients acquire or maintain the competencies they need to manage as well as possible their lives with a chronic disease. It is an integral and continuing part of patient care. It comprises organized activities, including psycho-social support, designed to make patients aware of and informed about their disease and about health care, hospital organization and procedures, and behavior related to health and disease, so that they (and their families) understand their disease and their treatment, collaborate with each other and take responsibility for their own care as a means of maintaining or improving their quality of life” [34].

TPE programs aim to empower patients to understand their disease and its treatment, develop self-monitoring capabilities, engage in self-care, and adapt therapy to their lifestyle [35].

Those programs typically involve four steps: conducting an educational diagnosis, establishing an educational contract, implementing the educational contract, and carrying out educational follow-up and reassessment if necessary [36]. “The educational diagnosis is the first step in the educational process and makes it possible to learn about the various aspects of the patient’s life and personality, to identify his/her needs, to evaluate his/her potential, and to take into account his/her requests and projects, with the aim of offering a personalized educational program” [37]. This step evaluates the patient’s health-related skills, encompassing health literacy (HL), according to the four levels delineated by Sorensen: being able to find, understand, evaluate health information, and apply and communicate it [38].

If we adopt a chronic disease approach to caries disease, it becomes possible to propose a TPE approach, where the patient is supported in managing their disease as an integral part of medical care. Thus, in an educational approach concerning caries disease with diet as a risk factor, the educational diagnosis should consider the patient’s level of oral health literacy and his/her psychological readiness for changing dietary behavior. Oral HL can be assessed using validated questionnaires, along with knowledge, attitude, and practice (KAP) questions related to oral health [39,40]. The patient’s psychological readiness for behavior change can be assessed using the five stages of change outlined in Prochaska and Di Clemente’s” transtheoretical model of behavioral change” [15,41]. Thus, when considering dietary measures associated with oral health, such as reducing sweetened soda intake, these five stages of change can be taken into account (Table 2).

Two cognitive processes associated with these stages of change are decisional balance, which refers to the patients’ perception of the advantages and disadvantages of behavior change, and self-efficacy, which reflects the patients’ belief in their ability to enact change in a particular action [42]. In the context of a therapeutic education consultation for patients with high cariosusceptibility, implementing a behavioral change theory allows for categorizing individuals based on their motivational or behavioral stage, helping to tailor strategies to the patient’s phase and perceived advantages and disadvantages of behavior change, as well as their self-efficacy feelings.

Here are some examples. For the perception of the benefit of modifying eating behavior, the patient might express, “Reducing my sugar intake will lead to fewer carious lesions, thus improving my oral health”. For the perception of the disadvantage of changing eating habits, the patient might express concerns such as “Besides giving up the sweet taste, adopting a healthier diet requires me to reorganize my lifestyle” or “I feel the need for sugar when I work at night”. For the feeling of self-efficacy, the patient may state “I feel confident in my ability to make dietary changes” or “I am unsure if I can handle this change: it seems too difficult for me”. These cognitive processes are crucial to consider, given that eating is a complex phenomenon encompassing both “a vital biological function and an essential social function” [43].

## 4. Future Directions

Therefore, careful observation and quantification of the patient’s eating habits are essential. The two key dietary measures in managing caries disease are the daily amount of sugar ingested (an amount exceeding 10E being associated with increased caries risk) and the frequency of intake (frequent sugar consumption being linked to maintaining a high acid pH and reduced buffering capacity) [44]. Nutritional education should be viewed as an interdisciplinary and multi-professional approach, addressing personal life balances and involving various factors [45]. Considering the dietary risk factors involved in caries disease implies directing preventive strategies towards understanding diet and adapting traditional nutritional discourse to real-world behavioral contexts [46]. Dietary objectives, such as reducing sugar consumption and managing intake frequency, should not be conflated with educational objectives, such as distinguishing cariogenic from non-cariogenic foods. 

According to Simon et al., these care objectives involve developing coping skills and are part of a broader set of psychosocial skills [45]. Dental practitioners must recognize that behavioral changes associated with health conditions should be approached incrementally within a motivational environment and adapted to the patient’s psychological skills and literacy. This approach is crucial for patient adherence, confidence, and commitment to change [46].

Then, if we consider another condition, such as diabetes, for instance, the management parallels that of caries disease in terms of dietary adjustments, despite the differing health issues involved. Managing diabetes shares similarities with caries disease in terms of dietary modifications, despite their distinct health issues. A study highlighted the influence of a patient’s familiar microenvironment on their perception of the disease and prevalence, indicating the significant role of support. The perception of dietary management in caries treatment is similarly affected by patient and practitioner attitudes toward nutritional education strategies [47].

An infra-cognitive dimension study demonstrated its potential interference with learning, suggesting that healthcare professionals may have limited mastery of scientific knowledge, hindering patient behavior change. Patient health literacy is crucial for treatment adherence and self-management, especially for evaluating received information [48]. Nutritional education practices should integrate both knowledge and emotional factors. Another study on dietary self-management practices of type 2 diabetic patients revealed individual beliefs about the effects of certain foods on glycemic control (each person has his or her own “beliefs” about the “harmful” effects of certain foods), and highlighted the influence of food availability and financial aspects. Therefore, practitioners must be mindful of patients’ dietary practices before implementing nutrition education and should integrate both knowledge and emotional factors [49].

**Figure 1:**
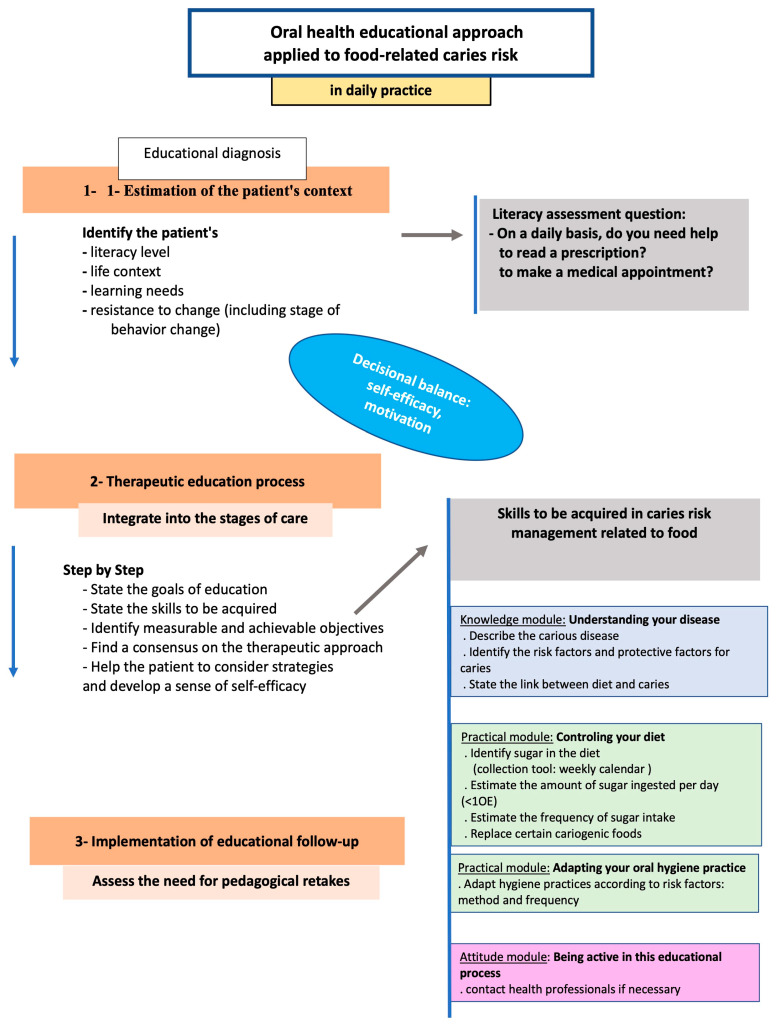
Educational approach to patients at risk of diet-related caries adapted to the daily practice of a dental practitioner.

Overall, these data led us to question the use of questionnaires to identify the caries risk of our patients. In most cases, they are used for research purposes or to document the medical record, but are of little use for the educational support needed to change dietary behavior. The questionnaires used appear to be disconnected from the treatment. This action remains a simple exchange of information in which the results are used to propose a change in eating habits without considering the necessary individual balance. Nonetheless, the use of such questionnaires can be an element of the diagnostic stage of any TPE program, making it possible to estimate educational needs at the same time. In particular, there are TPE programs on caries risk for the prevention of early caries in children [50]. However, we did not find any specific programs in the literature for adults. Based on the framework of TPE, we propose an example of an educational approach to patients at risk of diet-related caries, adapted to the dental practitioner’s practice [43] (Figure 1).

In daily practice, the integration of specific items associated with the caries risk assessment questionnaire would allow the educational diagnosis and assessment of the patient’s context. This part would include simple questions to concisely assess the level of literacy, the life context (isolated or surrounded), learning needs (knowledge, practices), and resistance to change. The latter is linked to the phases of acceptance of the disease (acceptance of the diagnosis: to be moderated by the context of the morbidity of caries), the stages of behavioral change (described in the Prochaska and Di Clemente cycle), and the patient’s health beliefs [41]. According to the Health Belief Model (HBM), to adhere to a treatment, the patient must be convinced that he or she is ill, that the proposed treatment can be beneficial, and that the benefits effectively outweigh the burdens, inconveniences, and efforts [51,52]. In the context of diet-related caries disease in adults, the skills to be acquired are of three kinds: knowledge (understanding one’s disease), practice (controlling one’s diet: quantity/frequency of sugar intake, adapting one’s hygiene practices), and attitude (being active and calling on professionals if necessary). The final evaluation stage, carried out some time later, will enable the practitioner to assess the skills acquired in relation to the state of health (activity of the carious disease: presence of new carious lesions/stability of the carious disease) and, if necessary, repeat the education.

## 5. Conclusions

In summary, despite the availability of various caries risk assessment tools incorporating dietary risk factors, there is a lack of educational tools associated with implementing therapy steps. The current approach remains prescriptive, limited to advice and information. Treating caries disease as a chronic disease necessitates a paradigm shift in care, akin to therapeutic patient education (TPE), by providing comprehensive patient support in disease management as an integral part of the medical care. Moreover, the pivotal role of dental practitioners in metabolic control underscores the significance of collaborating with other healthcare professionals, such as dieticians. This raises the question of the dental practitioner’s role in holistic patient care, and the nature of health education that could empower the patient, particularly with regard to dietary issues.

## Figures and Tables

**Table 1:** Tools for assessing eating behavior in the presence of caries disease among adult patients.

Name of Tool	Type of Tool	Food Parameters Measured
CAMBRA [16]	Hetero-questionnaire	Frequency of snacking (>three times daily)
Caries Care International [17]	Hetero-questionnaire	Frequency of free sugars; Amount of free sugars.
Caries Risk Assessment (CRA) [18]	Hetero-questionnaire	Frequency of sugars (primarily at mealtimes/frequent or prolonged between mealtimes/exposures day); Existence of eating disorders.
Cariogram [19]	Algorithm Hetero-questionnaire	Diet content (estimation of the cariogenicity of the food, in particular sugar content (diet history)); Frequency of snacking (questionnaire results, 24-h recall or dietary recall: 3 days).
Diet Assessment of Caries Risk [20]	Hetero-questionnaire	Frequency of snacking (<6/day or >6/day) and length of exposure (<15 min or 15–30 min or >30 min); Diet content (meal/snack structure); Amount of sugars (<12 ounces/day or 12–20 ounces/day or >20 ounces/day); Timing of sugars intake (with meals or with snacks or between meals/snacks); Drinking style (straw/open container/swishing within mouth).
International Caries Classification and Management System (ICCMS) [21]	Hetero-questionnaire	Frequency of sugar; Amount of sugar.
NNS FFQ (Non-Nutritive Sweetener Food Frequency Questionnaire) [22]	Self-questionnaire	Frequency (1x/week; 1x/week, 2–3x/week, 4–6x/week, 1x/day, 2x/day, 3x/day); Amount of sugars (<half a portion, one portion, one and a half portions, two portions, three or more portions).
Nutrition Questionnaire for Dental Caries Risk Factors [23]	Self-questionnaire	Thirteen questions and the patient should check all that apply: - Each day, when do you usually have drinks, including smoothies? - Each day, when do you usually have food? - When do you usually eat sugar or sweet foods such as cereals, cookies, cakes, baked goods, chocolate bars, fruit in syrup, or other sugary foods? - How often do you usually drink each of the following sugary drinks? - Do you add sugar to food or drinks including: white or brown sugar, honey, agave, other natural or processed syrup, and other natural sugars such as cane, coconut, or palm sugar? - Do you chew sugary gum? - How many servings of vegetables and fruits do you eat each day? - Is one serving of fruit one whole piece of fruit or half a cup of chopped or frozen fruit? - How often do you usually drink the following types of milk? - If you drink soy milk or other milk, has it been fortified with vitamins and minerals? - When do you usually have milk? - How often do you usually eat cheese or yogurt? - When do you usually have cheese or yogurt?
24 H recall [24]	Food diary	Patients’ dietary information over the last 24 h.

**Table 2:** Transtheoretical model of behavioral change associated with caries risk eating behavior.

Stages of Change Defined by Prochaska and Di Clemente [41]	Definition of the Stage of Change	Example in the Presence of a Caries Risk Eating Behavior (Soda Consumption)
1—Pre-contemplation	The patient is not involved. The patient does not consider any action in favor of a change in behavior within six months.	The patient does not change sugar intake.
2—Contemplation	The patient is concerned and heeds the information. He/she expresses the intention to change his/her behavior in the next six months and may take a first step.	The patient agrees to complete a weekly sugar drink diary to estimate the daily consumption of sugar.
3—Preparation	The patient prepares for the action-decision to change: he/she intends to act in the short term (within 30 days) with a stated realistic plan.	The patient speaks in favor of decreasing the frequency of soda intake.
4—Action	Initiation of change: implementation of a new behavior: The patient has concretely engaged in observable actions to modify his/her behavior during the last six months. These actions must be sufficient to promote health and be maintained over time.	The patient chooses sugar-free sodas and implements a gradual and quantifiable reduction in intake: only during meals, then only one per day, then one per week, and then reserved for festive moments... and uses a straw. At this stage, it must be encouraged.
5—Relapse	Maintaining the change. Maintainence of his/her behavior for a period of more than six months, with vigilance for relapse.	The patient comes to the follow-up consultations over a long period of time and maintains the new oral health behaviors.

## Data Availability

No new data were created or analyzed in this study. Data sharing is not applicable to this article.
